# Koala Retrovirus in Northern Australia Shows a Mixture of Stable Endogenization and Exogenous Lineage Diversification within Fragmented Koala Populations

**DOI:** 10.1128/JVI.02084-20

**Published:** 2021-03-10

**Authors:** Bonnie L. Quigley, Alistair Melzer, William Ellis, Galit Tzipori, Karen Nilsson, Olusola Olagoke, Amy Robbins, Jonathan Hanger, Peter Timms

**Affiliations:** a GeneCology Research Centre, University of the Sunshine Coast, Sippy Downs, Queensland, Australia; b CQUniversity, Koala Research-CQ, School of Medical and Applied Sciences, Rockhampton, Queensland, Australia; c School of Agriculture and Food Science, The University of Queensland, Brisbane, Queensland, Australia; d Lone Pine Koala Sanctuary, Fig Tree Pocket, Queensland, Australia; e Endeavour Veterinary Ecology, Toorbul, Queensland, Australia; Icahn School of Medicine at Mount Sinai

**Keywords:** koala, koala retrovirus, KoRV, Queensland, exogenous, endogenous

## Abstract

The koala population in northern Australia has become increasingly fragmented due to natural and human-made barriers and interventions. This situation has created a unique opportunity to study both endogenous and exogenous koala retrovirus (KoRV). To determine the impact that population isolation has had on KoRV diversity in Queensland, 272 koalas from six fragmented koala populations were profiled for their KoRV provirus across two natural biogeographical barriers (the St. Lawrence Gap and the Brisbane Valley Barrier), one humans-made geographical barrier (the city of Brisbane), and two translocation events (the single movement of koalas to an island and the repeated movement of koalas into a koala sanctuary). Analysis revealed that all koalas tested were KoRV-A positive, with 90 to 96% of the detected KoRV provirus from each koala representing a single, likely endogenous, KoRV-A strain. The next most abundant proviral sequence was a defective variant of the dominant KoRV-A strain, accounting for 3 to 10% of detected provirus. The remaining KoRV provirus represented expected exogenous strains of KoRV and included geographically localized patterns of KoRV-B, -C, -D, -F, -G, and -I. These results indicate that lineage diversification of exogenous KoRV is actively ongoing. In addition, comparison of KoRV provirus within known dam-sire-joey family groups from the koala sanctuary revealed that joeys consistently had KoRV proviral patterns more similar to their dams than their sires in KoRV-B, -C, and -D provirus composition. Collectively, this study highlights both the consistency of endogenous KoRV and the diversity of exogenous KoRV across the fragmented koala populations in northern Australia.

**IMPORTANCE** KoRV infection has become a permanent part of koalas in northern Australia. With KoRV presence and abundance linked to more severe chlamydial disease and neoplasia in these koalas, understanding how KoRV exists throughout an increasingly fragmented koala population is a key first step in designing conservation and management strategies. This survey of KoRV provirus in Queensland koalas indicates that endogenous KoRV provirus is ubiquitous and consistent throughout the state, while exogenous KoRV provirus is diverse and distinct in fragmented koala populations. Understanding the prevalence and impact of both endogenous and exogenous KoRV will be needed to ensure a future for all koala populations.

## Abstract (precis)

KoRV infection has become a permanent part of koalas in northern Australia. With KoRV presence and abundance linked to more severe chlamydial disease and neoplasia in these koalas, understanding how KoRV exists throughout an increasingly fragmented koala population is a key first step in designing conservation and management strategies.

## INTRODUCTION

Koala retrovirus (KoRV) is found both endogenously and exogenously in koalas (Phascolarctos cinereus) (1, 2). It is hypothesized that KoRV entered the koala population in Queensland (the northernmost part of their Australian range) from a spill-over event from either a rodent or bat source (3, 4) and has since adapted to its new marsupial host. Since that original introduction at an unknown historical time, KoRV has begun endogenizing or permanently incorporating its provirus into koala germ line genomes in the northern Australian koala population (5). Examination of KoRV long terminal repeats (LTRs) estimates that KoRV endogenous integration began no more than 49,900 years ago, although a much more recent time of integration is possible (6). In parallel, KoRV has diversified into nine recognized subtypes (KoRV-A to -I), based on differences in the receptor binding domain region of the envelope gene (7–10). Within Queensland koalas, full koala genome sequencing, historical koala skin examination, and field studies of KoRV provirus in wild koala populations have strongly suggested that the KoRV-A subtype contains both endogenous (vertically transmitted, based on high-copy [apparently germ line] proviral insertion) and exogenous (horizontally transmitted, based on various strain types) strains, while KoRV-B to -I subtypes appear to contain only exogenous strains (based on low-copy-number [apparently somatic] insertions) (11–14). This unique set of circumstances has made Queensland koalas a very interesting and valuable population in which to study KoRV dynamics.

**FIG 1:**
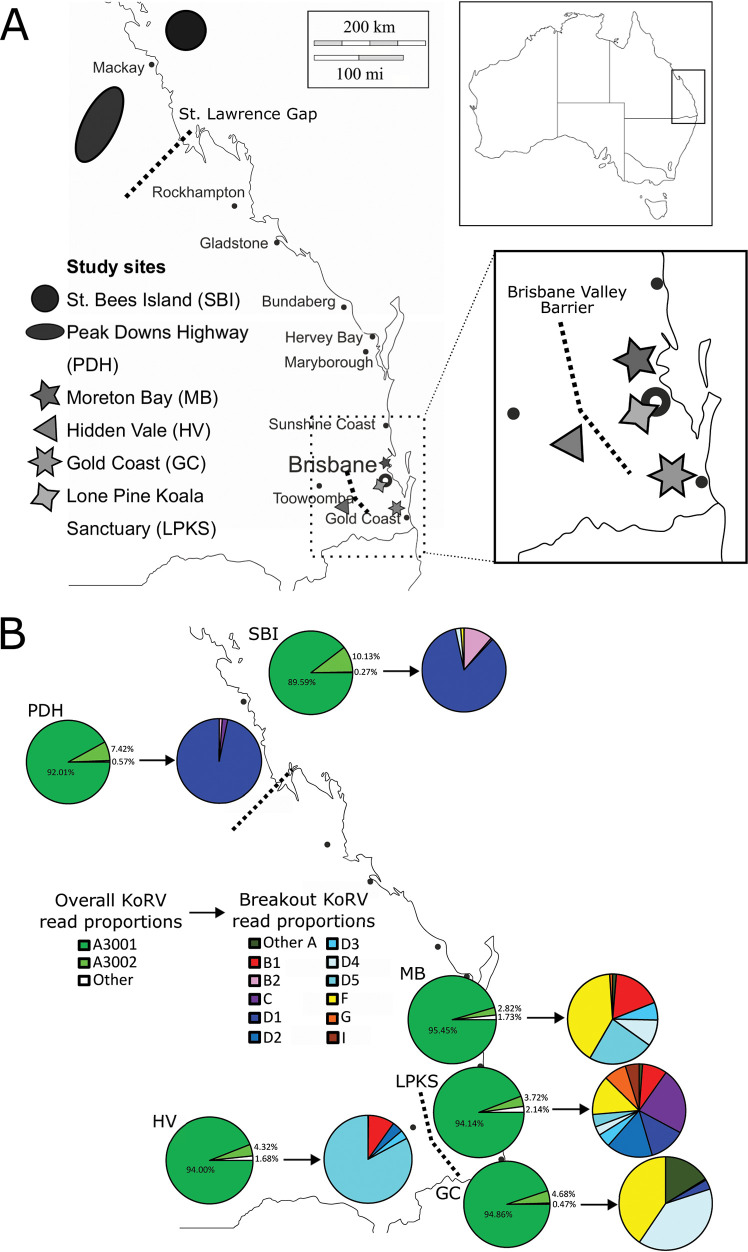
KoRV proviral diversity detected across Queensland. (A) Locations of koala sampling sites surveyed, with local cities and reported biogeographical barriers (St. Lawrence Gap and Brisbane Valley Barrier) indicated on the map. (B) Average KoRV proviral reads per koala detected in each region. For each study region, the first pie represents the overall average KoRV proviral reads (KoRV-A strain A3001, A3002, and all other strains), while the second pie is an expansion of the “all other strains” section, colored by KoRV subtype and clade detected.

Despite not knowing when KoRV entered the Queensland koala population, there are several Queensland features that have led to koala population fragmentation that do have known dates. Starting on a geological scale, there are two major biogeographical barriers that are believed to have impacted koala populations (15). The St. Lawrence Gap, located along the Queensland central east coast between the cities of Mackay and Rockhampton, and the Brisbane Valley Barrier, located in the southeast corner of Queensland west of Brisbane, are two regions where traditional coastal eucalyptus forests give way to open woodland and savannah or grassland regions, respectively (Fig. 1A). The St. Lawrence Gap has also been noted as a drought- and fire-prone area, making this a hard landscape for koalas. These conditions are believed to have limited koala movement across these regions and have been recognized as biogeographical barriers since the Pleistocene period (2.5 million to 11.5 thousand years ago) (15). More recently, the city of Brisbane was established in 1825 (16), which would have begun limiting the movements of koalas around that southeast corner of Queensland (Fig. 1A). In 1927, Lone Pine Koala Sanctuary was established within the city of Brisbane. Recognized as the largest and oldest koala sanctuary in the world, this facility has been housing and breeding mostly Queensland koalas in close contact for almost 100 years, creating a uniquely isolated captive koala population (17). Finally, a new, isolated island population of koalas was established on St. Bees Island (just offshore from Mackay in central east Queensland) from neighboring mainland populations in the 1930s (Fig. 1A) (18). These various translocations and natural or human-made barriers have fragmented several Queensland koala populations, causing groups of koalas to be isolated from each other for as little as 90 years up to possibly thousands or hundreds of thousands of years. As such, Queensland has created a unique landscape in which to compare KoRV diversity across different fragmented koala populations.

This study compares the diversity of KoRV provirus detected in 272 koalas from six distinct Queensland koala populations, spanning two natural biogeographical barriers, one isolated island population and one long-term captive koala colony. The goal was to determine the impact, if any, these short- or long-term separation events/barriers have had on KoRV diversity across Queensland. Interestingly, KoRV provirus profiling revealed that endogenous KoRV was virtually identical and by far the most dominant KoRV strain across the entire state, with only minor contributions of different exogenous KoRV strains contributing a unique KoRV pattern to each fragmented koala population. Additionally, the long-term captive koala colony contained the most KoRV diversity within Queensland, allowing for a comparison of KoRV provirus profiles within dam-sire-joey family units. This detailed family comparison revealed that exogenous KoRV patterns detected in joeys were much more related to their dam’s KoRV profile than their sire’s KoRV profile, further strengthening a proposed link in dam-to-joey exogenous KoRV transmission.

## RESULTS

### Generating KoRV provirus profiles across Queensland.

To assess KoRV diversity across central east to southeast Queensland, samples from a total of 272 koalas were collected from six study sites (Fig. 1A). These included tissue samples from St. Bees Island (*n *=* *30), scat samples from Peak Downs Highway (*n *=* *39), and blood samples from koala populations at Moreton Bay (*n *=* *62), Hidden Vale (*n *=* *20), Gold Coast (*n *=* *39), and Lone Pine Koala Sanctuary (*n *=* *82). Previous study has determined that KoRV proviral profiles generated from multiple sample types can be combined for analysis (12), so all samples were DNA extracted, PCR amplified to obtain the KoRV envelope gene receptor binding region from all subtypes simultaneously, deep amplicon sequenced, and processed to obtain representative operational taxonomic units (OTUs) or strains present in each koala. This procedure is analogous to several previous KoRV proviral studies (7, 11, 12, 19, 20). Study samples generated an average of 188,016 ± 69,301 KoRV proviral reads, with an overall average Good’s coverage estimate of 0.999996, meaning that overall, each sample was predicted to have been profiled to 99.9996% completion. Detailed KoRV provirus profiles for each koala can be found in Table S1 in the supplemental material.

### Overall KoRV proviral diversity in Queensland.

This Queensland data set contained 208 distinct KoRV proviral strains, representing seven established KoRV subtypes. These included 22 KoRV-A strains, 18 KoRV-B strains, 12 KoRV-C strains, 111 KoRV-D strains, 31 KoRV-F strains, 6 KoRV-G strains, and 8 KoRV-I strains. No KoRV-E or KoRV-H strains were detected from these koalas. Of note, this data set is the first to report KoRV-C strains in Australia. Previous reports of KoRV-C have been from Japanese zoo koalas (8, 21), where zoo animals were obtained from Lone Pine Koala Sanctuary (internal sanctuary records).

**FIG 2:**
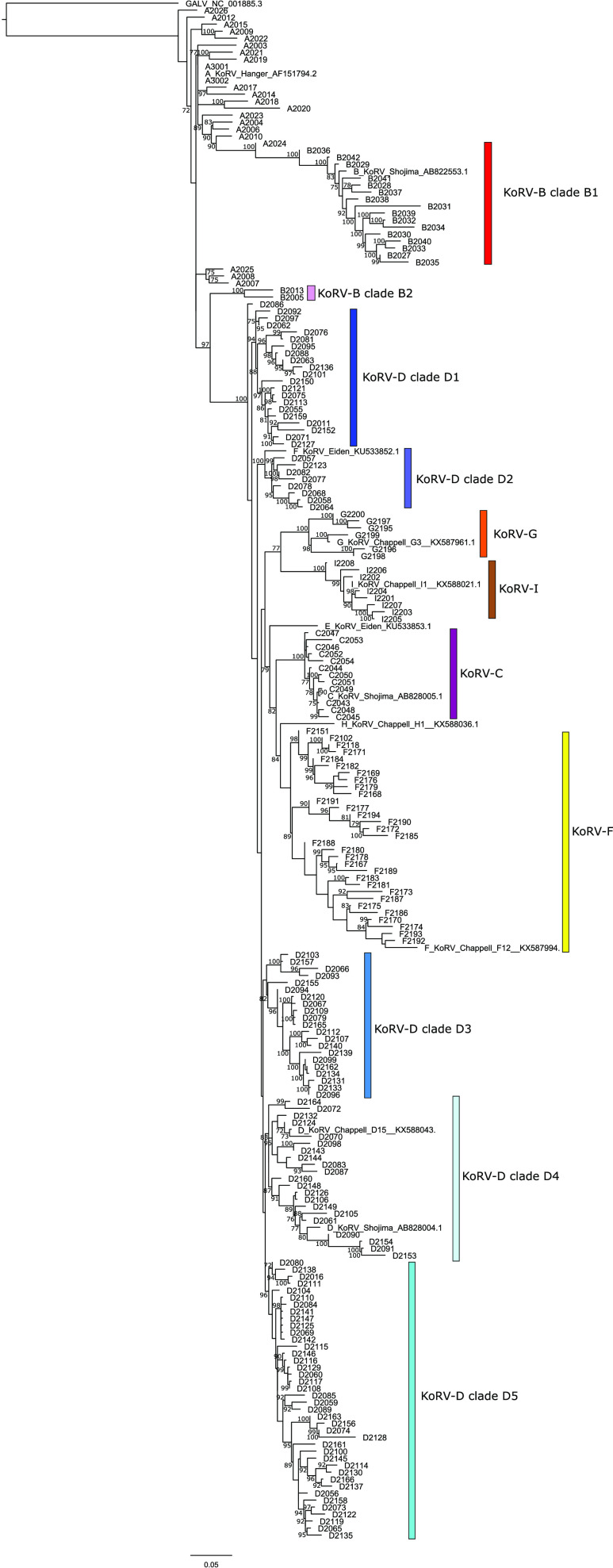
Maximum likelihood phylogeny of all the Queensland KoRV proviral OTUs detected. Bootstrap values greater than 70 are indicated. Reference strains are indicated by the original publishing author’s name and accession number and include Hanger et al. 2000 (original KoRV/KoRV-A), Shojima et al. 2013 (KoRV-B/J = OJ-4, 11-4; KoRV-C = OJ-4, 11-2; KoRV-D = OJ-4, 11-1), Xu et al. 2015 (Eiden KoRV-E and KoRV-F), and Chappell et al. 2017 (KoRV-D = D15, KoRV-F = F12, KoRV-G = G3, KoRV-H = H1, KoRV-I = I1). Gibbon ape leukemia virus (GALV) was included as an outgroup.

Phylogenetic analysis of the KoRV proviral strains detected in Queensland revealed relationships consistent with previous KoRV studies (Fig. 2) (7, 12, 19). KoRV-B strains separated into two distinct clades, clade B1 and B2, similar to a previous study of KoRV provirus across Australia (12). KoRV-D also separated into five well-supported clades, clades D1 to D5, also expected for this paraphyletic subtype (7, 12, 19). The other KoRV subtypes also grouped as expected, with KoRV-A representing the closest subtype to other retroviruses, like gibbon ape leukemia virus (GALV), and KoRV-C, -F, -G and -I branching as distinct monophyletic clades within the overall KoRV-D branch (Fig. 2) (7).

### Similarities in endogenous KoRV provirus across study regions.

All koalas in Queensland possessed two KoRV-A proviral strains, designated A3001 and A3002 (Fig. 1B). Strain A3001 is identical to the originally reported KoRV strain by Hanger et al. in 2000 (2), and a recent study has strongly suggested that this is the endogenous strain in Australian koalas (12). Across the Queensland study sites, KoRV-A strain A3001 represented an average of 89.6% to 95.5% of all KoRV proviral reads (Fig. 1B). This supports the assertion that A3001 is the endogenous KoRV strain in Australia and indicates that 90 to 96% of all KoRV provirus in Queensland is represented by a single strain. Additionally, KoRV-A strain A3002 was also detected in every Queensland koala tested and represents a defective variant of A3001, with a 2-bp insertion (at positions 218 to 219 of the amplicon) that leads to a frameshift and stop codon in the middle of the envelope gene sequence. KoRV-A strain A3002 represented an average of 2.8% to 10.1% of all KoRV proviral reads across the study sites (Fig. 1B). Together, these two KoRV-A strains (A3001 and its defective version, A3002) were found to account for 97.9% to 99.5% of all the KoRV provirus detected in Queensland.

### Differences in exogenous KoRV provirus across study regions.

The remaining small proportion of non-A3001/A3002 KoRV proviral reads detected across Queensland appeared to represent the exogenous KoRV strains present in the koala population. Despite composing only a small fraction of the total KoRV provirus present in koalas, these 206 strains represented an impressive range of lineage diversification and clearly revealed KoRV distinctions between study sites (Fig. 1B, Table 1). Overall, Lone Pine Koala Sanctuary had the most KoRV subtype prevalence beyond KoRV-A, with most koalas positive for KoRV-B (96%) and KoRV-D (99%), over two-thirds positive for KoRV-C (72%), and over one-third positive for KoRV-F (41%), KoRV-G (38%) and KoRV-I (34%) (Table 1). At the other extreme, koalas in the Peak Downs Highway region possessed low KoRV prevalence beyond KoRV-A, with koalas only positive for KoRV-D clade D1 (97%) and the occasional koala also positive for KoRV-B (8%) and/or KoRV-C (10%) (Table 1).

**TABLE 1:** Percentage of koalas positive for each KoRV subtype/clade by region

Subtype/clade	% Positive koalas in^*a*^:					
	SBI (*n*= 30)	PDH (*n* = 39)	MB (*n* = 62)	HV (*n* = 20)	GC (*n* = 39)	LPKS (*n* = 82)
KoRV-A	100	100	100	100	100	100
KoRV-B	53	8	34	40	26	96
B1	0	0	34	40	3	96
B2	53	8	0	0	23	0
KoRV-C	20	10	0	0	0	72
KoRV-D	97	97	97	100	64	99
D1	97	97	0	0	28	33
D2	0	0	0	10	0	76
D3	0	0	37	20	0	27
D4	20	0	29	0	62	9
D5	0	0	60	90	0	16
KoRV-F	20	0	66	15	85	41
KoRV-G	0	0	3	0	0	38
KoRV-I	0	0	0	0	0	34

a SBI, St. Bees Island; PDH, Peak Downs Highway; HV, Hidden Vale; MB, Moreton Bay; GC, Gold Coast; LPKS, Lone Pine Koala Sanctuary.

By subtype, after KoRV-A, KoRV-D had the highest overall prevalence at the different study sites (64% to 100%), with the five KoRV-D clades having distinct distributions around the state (Table 1). KoRV-B was the only other subtype detected at all studies sites; however, only Gold Coast koalas possessed both clade B1 and B2, with clade B1 otherwise limited to the sites around Brisbane (Moreton Bay, Hidden Vale and Lone Pine Koala Sanctuary) and clade 2 limited to central east Queensland (St. Bees Island and Peak Downs Highway) (Fig. 1B, Table 1). KoRV-F ranged in prevalence around the state, with only the Peak Downs Highway koala population lacking this subtype. KoRV-C was only detectable at a low prevalence in central east Queensland (St. Bees Island and Peak Downs Highway), with a higher prevalence at Lone Pine Koala Sanctuary. Finally, KoRV-G and KoRV-H were primarily detected at Lone Pine Koala Sanctuary, with a very low level of KoRV-G also detected in Moreton Bay (Table 1).

### Overall comparison of KoRV proviral profiles.

**FIG 3:**
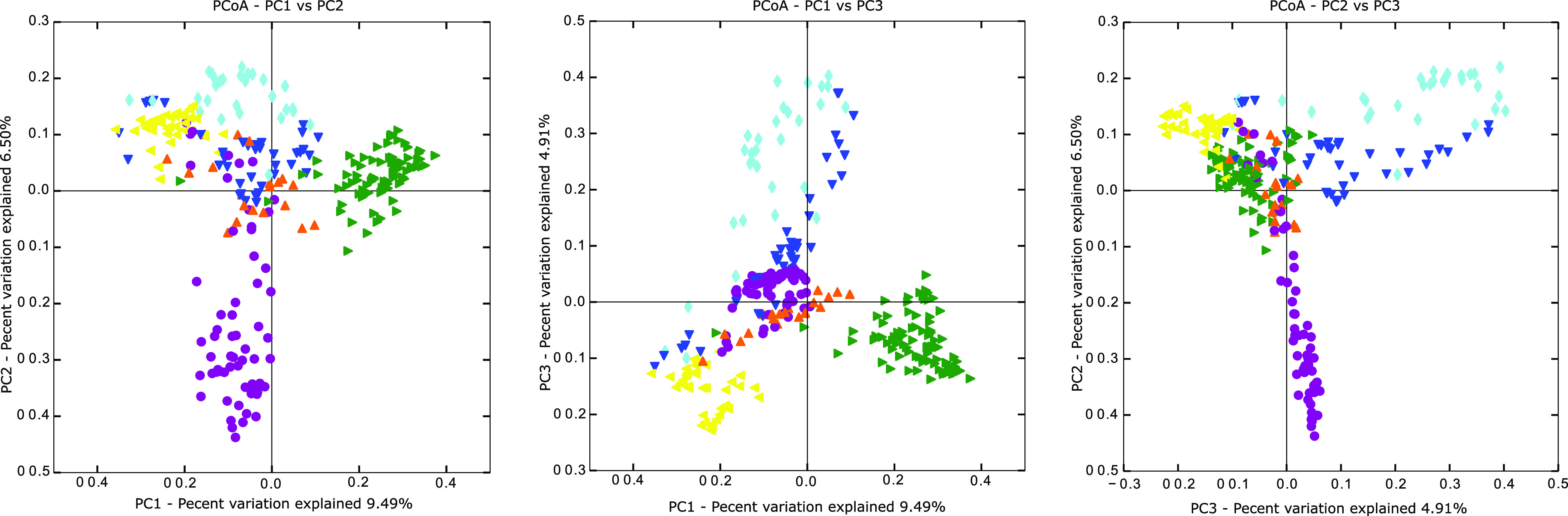
Principal coordinate analysis (PCoA) of total koala KoRV proviral profiles by region. Regions are as indicated: St. Bees Island (SBI), light blue diamonds; Peak Downs Highway (PDH), yellow triangles, left facing; Hidden Vale (HV), orange triangles, upright; Moreton Bay (MB), purple circles; Gold Coast (GC), dark blue triangles, bottom facing; and Lone Pine Koala Sanctuary (LPKS), green triangles, right facing.

Principal coordinate analysis (PCoA) of total KoRV proviral profiles continued to highlight the differences exogenous KoRV contributed to each study region, with total koala profiles clustering distinctly by study area (Fig. 3). Unexpectedly, St. Bees Island and Gold Coast koalas grouped closely in this analysis (Fig. 3). Although not initially apparent when examined by average reads per koala per region (Fig. 1B), examination of subtype and clade prevalence between St. Bees Island and Gold Coast does suggest similarities between sites (Table 1).

### KoRV proviral profiles within dam-sire-joey family groups.

**FIG 4:**
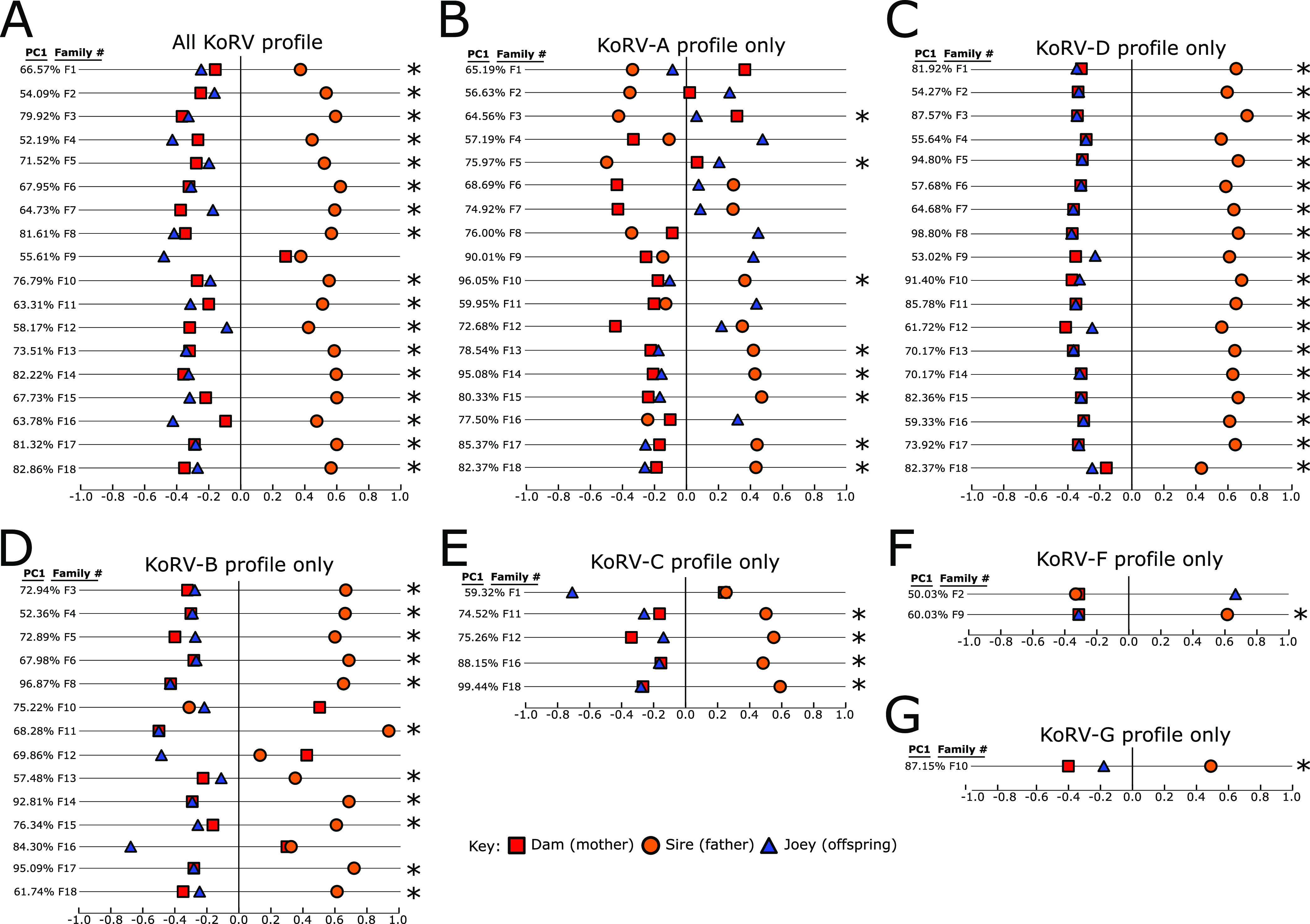
First dimension of a principal coordinate analysis (PCoA) of specific KoRV proviral profiles from family units (dam-sire-joey) at Lone Pine Koala Sanctuary. Panels represent the comparison of total KoRV provirus (A), KoRV-A provirus only (B), KoRV-D provirus only (C), KoRV-B provirus only (D), KoRV-C provirus only (E), KoRV-F provirus only (F), and KoRV-G provirus only (G). Only family units where all three members (dam, sire, and joey) were positive for the KoRV subtype indicated were included. The percent variation in proviral profiles explained by this first dimension is indicated in the PC1 column to the left of the family unit number. Family units where the joey’s KoRV provirus profile is closer to their dam’s profile (versus their sire’s profile) are indicated with an asterisk.

Lone Pine Koala Sanctuary has an active breeding program, resulting in many well-documented family units (dam-sire-joey) at the time of this study. The high prevalence of multiple KoRV subtypes detected at the sanctuary, together with 18 complete family units represented in the sanctuary sample collection, allowed for direct comparison of KoRV proviral patterns between joeys and their parents. A list of the koala family units analyzed can be found in Table S2. PCoA analysis compared both the presence and abundance of KoRV strains detected within each dam, sire, and joey family unit and calculated a distance metric to represent how similar or different the profiles were. The first component of this metric, representing the largest component of the distance between samples, is diagrammed in Fig. 4, showing the relationship between dam-sire-joey KoRV proviral patterns by either total KoRV provirus (Fig. 4A) or subsets of KoRV subtype-specific provirus (Fig. 4B to G). Only family units where all three members were positive for the subtype being analyzed were included.

Comparisons revealed that joeys most often had proviral patterns more similar to their dams than their sires in overall total KoRV profiles (94% [17/18]; Fig. 4A) and exogenous KoRV profiles (KoRV-B, 75% [9/12], KoRV-C, 80% [4/5], KoRV-D, 100% [18/18], KoRV-F, 50% [1/2] and KoRV-G, 100% [1/1]; Fig. 4C to G). This contrasted with the mixed endogenous and exogenous KoRV-A profiles, where only 44% (8/18) of family units had joey proviral patterns more similar to their dams than their sires (Fig. 4B).

## DISCUSSION

Koala populations in Queensland, Australia, have been fragmented by several natural and human-made factors. Historically, constraints on koala habitat suitability around the St. Lawrence Gap and Brisbane Valley Barrier have had the potential to separate central east Queensland and southeast Queensland koala populations for thousands of years or more. More recently, human settlement, habitat clearing, and deliberate koala translocations in the last 90 to 200 years have separated koala populations in the state by a major city and relocated animals to confined sanctuary and island locations. Combined, these factors have resulted in several isolated, fragmented koala populations along the Queensland coast. While challenging from a conservation and wildlife management perspective, this situation has created a unique opportunity to examine the diversification of a retrovirus in the koala population undergoing active endogenization in this setting.

Although it is not known when KoRV first entered the koala population, it has been estimated that KoRV endogenization into the koala germ line genome began within the last 49,900 years (6). The strain most likely representing the endogenizing KoRV-A strain, A3001 (12), was detected as the dominant provirus at a relatively consistent level in every koala tested across all six fragmented Queensland koala populations surveyed. This result suggests that the two natural biogeographical barriers examined in this survey, the St. Lawrence Gap and Brisbane Valley Barrier, have not had an appreciable impact on the initial spread or endogenization of KoRV across Queensland. This finding was not completely unexpected, as the St. Lawrence Gap may have opened and closed with climate shifts over time, koalas have the capacity to move relatively long distances through open forest and savannah, and genetic analysis has shown some evidence of potential mixing between Queensland koalas (22, 23). The consistency of endogenous KoRV in koala populations more recently fragmented/translocated by human activities also suggests that significant endogenization predates the last 90 to 200 years. Overall, this study found that the majority of KoRV provirus in Queensland koalas is consistently represented by one strain of KoRV (A3001), with the remaining KoRV provirus in Queensland made up by a defective variant of the dominant strain (A3002) and low levels of additional exogenous variants.

While the exogenous KoRV provirus detected in this study represented a small proportion of the overall KoRV provirus in Queensland, this fraction contained impressive diversity and clear evidence of linage diversification in each of the isolated, fragmented koala populations. In central east Queensland, 90 years of separation generated modest differences between the St. Bees Island and Peak Downs Highway koala populations, with similarities still evident in their KoRV-B clade B2, KoRV-C, and KoRV-D clade D1 strains. Moving to the southeast corner of Queensland, KoRV-B clade B1, KoRV-D clades D3 and D4, and KoRV-F had similarities between the Moreton Bay, Hidden Vale, and Gold Coast koala populations, with each population having variations making their proviral profiles distinct. Finally, Lone Pine Koala Sanctuary was distinct from all the wild populations, appearing as a mixing pot of all the variation seen in the wild. Missing only KoRV-B clade B2, Lone Pine koalas possessed every other major subtype or clade of KoRV provirus detected across Queensland. This suggests that almost 100 years of close contact and breeding of Queensland koalas at this sanctuary has created an ideal environment to maintain and diversify KoRV. Taken together, this survey suggests that lineage diversification of KoRV is still an active process in Queensland. and koalas separated by as little as 90 to 200 years can generate distinct population shifts in their KoRV proviral diversity.

Finally, the unique KoRV prevalence and diversity at Lone Pine Koala Sanctuary allowed for additional analysis of how KoRV proviral patterns compared between parents and offspring. Previous studies have detected strong correlations between KoRV-B-positive dams producing KoRV-B-positive joeys (9, 24), with other study indicating the KoRV-B dam-joey transmission link is not universal (21). Interestingly, no correlation has been detected between KoRV-D or KoRV-F positive dams and their joeys (10). The deep-sequencing approach taken in this study generates a more sensitive measure of provirus within a koala compared to previous PCR approaches and offered a new perspective on the relationship between KoRV infection in dams, sires, and joeys. From the 18 family groups present at Lone Pine Koala Sanctuary, joeys had KoRV-B, KoRV-C, and KoRV-D provirus profiles more similar to those of their dams most or all of the time (75%, 80%, and 100% of families, respectively) compared to the profiles of their sires. It has been hypothesized that the close contact between dam and joey while the joey is in the pouch, along with KoRV proteins found in dam’s milk (25), makes the dam the most influential transmission source for exogenous KoRV strains to a joey. Profiles from these Lone Pine family units support this theory and suggest the KoRV status of the dam has a much larger impact on the joey’s KoRV status than their sire, regardless of subtype. However, there were family groups that did not have closely related dam-joey KoRV-B, KoRV-C, and KoRV-F provirus profiles, suggesting that exogenous KoRV transmission cannot be entirely explained by dam-joey close contact and dam’s milk alone. Interestingly, given the high background level of endogenous KoRV-A provirus in all koalas, a dam-joey connection was not detected in the few exogenous KoRV-A strains existing in these families.

KoRV infection has become a permanent part of koalas in northern Australia. With evidence that the presence of some subtypes can lead to negative health outcomes in koalas (9, 24, 26), understanding how KoRV exists throughout the koala population is a key first step in designing conservation and management strategies. This survey of KoRV provirus in Queensland koalas indicates that endogenous KoRV provirus is ubiquitous and consistent throughout the state but that exogenous KoRV provirus is diverse and distinct in fragmented koala populations. As our understanding of the impacts of both endogenous and exogenous KoRV expands, the future of these fragmented koala populations will become clearer.

## MATERIALS AND METHODS

### Sample collection and processing.

Tissue and scat samples were opportunistically collected from St. Bees Island koalas (*n *=* *30) and koalas in the vicinity of Peak Downs Highway, Queensland (*n *=* *39), respectively. Blood samples from Hidden Vale koalas (*n *=* *20) and Moreton Bay koalas (*n *=* *62) were collected as part of an ongoing population-wide health management program (University of the Sunshine Coast [USC] animal ethics number ANA1380; Queensland Government Scientific Purposes Permit WISP11532912) and have been previously reported (20). Blood samples from Gold Coast koalas (*n *=* *39) and Lone Pine Koala Sanctuary koalas were collected as part of routine veterinary care (USC animal ethics number ANE1942). All samples were DNA extracted using the QIAamp DNA minikit (Qiagen) by following the manufacturer’s protocol for each sample type.

### KoRV *env* proviral amplicon generation and sequencing.

KoRV *env* amplicons spanning the receptor binding domain of the *env* gene (positions 22 to 514 of the KoRV-A *env* gene) were generated with the primers env22.F (TCGTCGGCAGCGTCAGATGTGTATAAGAGACAGGCTTCTCATCTCAAACCCG-CGCC) and env514.R (GTCTCGTGGGCTCGGAGATGTGTATAAGAGACAGGGGTTGCCAGTAGGCGGTTCC) according to previous studies (7, 11, 24). Briefly, HotStarTaq plus master mix (Qiagen), 0.2 μM env22.F, 0.2 μM env514R, and 1 μl of sample DNA were combined for amplification at 95°C for 15 min, 40 cycles of 95°C for 30 s, 55°C for 30 s, and 72°C for 30 s, and a final step at 72°C for 5 min. Amplicons were sequenced (MiSeq; Illumina) using the V3 300-bp paired-end chemistry after barcoding (eight rounds of tag addition amplification) at Ramaciotti Centre for Genomics (Sydney, Australia).

### KoRV OTU generation.

KoRV sequence reads were filtered for length and primer trimmed with cutadapt (27) before forward and reverse amplicon reads were merged with FLASH (28). Operational taxonomic units (OTUs) were generated with the QIIME 1.9.1 software package (29) with the UCLUST algorithm (set to 97% sequence identity) and USEARCH algorithm (set to 99% minimum percent identity of match over at least 98% of both reference and read) (30). This generated a KoRV OTU frequency table (see Table S1 in the supplemental material). OTUs that contained fewer than 100 reads (across all samples) were removed from the data set, and the remaining OTUs were BLAST searched (31) against a library of known KoRV *env* sequences (generated from references 7 and 24) for initial identification. KoRV subtype was further refined based on taxonomic placement of each OTU in a phylogenetic tree.

### Maximum likelihood phylogeny.

A maximum likelihood phylogenetic tree was generated using KoRV OTU DNA sequences aligned with mafft (32) before ModelFinder determined the best fit model (K3P+R5) based on Bayesian information criterion (33), and IQ-TREE (34) and UFBoot2 (35) constructed the tree with 1,000 bootstrap replicates.

### PCoA.

To compare the KoRV OTU profiles between koalas, the QIIME 1.9.1 software package (29) was used to first calculate beta diversity Canberra distances, followed by principal coordinate analysis (PCoA). PCoA plots were generated based on total KoRV OTU profiles as well as profiles divided by KoRV subtype.

### Data availability.

Sequences of the OTU generated in this study can be found under the GenBank accession numbers MW122516–MW122721.

## Supplementary Material

Supplemental file 1
